# Kurarinone Attenuates BLM-Induced Pulmonary Fibrosis via Inhibiting TGF-β Signaling Pathways

**DOI:** 10.3390/ijms22168388

**Published:** 2021-08-04

**Authors:** Soo-Jin Park, Tae-hyoun Kim, Kiram Lee, Min-Ah Kang, Hyun-Jae Jang, Hyung-Won Ryu, Sei-Ryang Oh, Hyun-Jun Lee

**Affiliations:** 1 Natural Medicine Research Center, Korea Research Institute of Bioscience and Biotechnology (KRIBB), 30 Yeongudanji-ro, Ochang-eup, Cheongwon-gu, Cheongju, Chungbuk 28116, Korea; soojin921@kribb.re.kr (S.-J.P.); whitevet81@gmail.com (T.-h.K.); dlrlfka05@hanmail.net (K.L.); alsdk0502@kribb.re.kr (M.-A.K.); water815@kribb.re.kr (H.-J.J.); ryuhw@kribb.re.kr (H.-W.R.); seiryang@kribb.re.kr (S.-R.O.); 2 Department of Biomolecular Science, University of Science & Technology (UST), Daejeon 341113, Korea

**Keywords:** kurarinone, pulmonary fibrosis, TGF-β, epithelial–mesenchymal transition

## Abstract

Idiopathic pulmonary fibrosis (IPF) is a refractory interstitial lung disease for which there is no effective treatment. Although the pathogenesis of IPF is not fully understood, TGF-β and epithelial–mesenchymal transition (EMT) have been shown to be involved in the fibrotic changes of lung tissues. Kurarinone is a prenylated flavonoid isolated from *Sophora Flavescens* with antioxidant and anti-inflammatory properties. In this study, we investigated the effect of kurarinone on pulmonary fibrosis. Kurarinone suppressed the TGF-β-induced EMT of lung epithelial cells. To assess the therapeutic effects of kurarinone in bleomycin (BLM)-induced pulmonary fibrosis, mice were treated with kurarinone daily for 2 weeks starting 7 days after BLM instillation. Oral administration of kurarinone attenuated the fibrotic changes of lung tissues, including accumulation of collagen and improved mechanical pulmonary functions. Mechanistically, kurarinone suppressed phosphorylation of Smad2/3 and AKT induced by TGF-β1 in lung epithelial cells, as well as in lung tissues treated with BLM. Taken together, these results suggest that kurarinone has a therapeutic effect on pulmonary fibrosis via suppressing TGF-β signaling pathways and may be a novel drug candidate for pulmonary fibrosis.

## 1. Introduction

Idiopathic pulmonary fibrosis (IPF) is an interstitial lung disease associated with chronic inflammation, fibrotic change of lung tissues and impaired lung function [1]. Due to the irreversibility of pulmonary fibrosis, IPF patients show uniquely poor prognosis and a low median survival rate of 3 years [2]. There are only two drugs, pirfenidone (PFD) and nintedanib, that have been approved for pulmonary fibrosis, and they are effective in improving lung functions and reducing mortality. However, they cannot completely cure the disease [3]. Therefore, there is an urgent need to develop new therapeutic agents for pulmonary fibrosis.

Although the pathogenesis of pulmonary fibrosis is not yet fully understood, dysregulation of wound healing processes induced by persistent damage to lung epithelial cells has been considered as a trigger of IPF [4]. The development of pulmonary fibrosis involves lung injury, inflammation, myofibroblast formation and accumulation of extracellular matrix (ECM). In injured lung epithelium, TGF-β causes clot formation and production of PAI-1, IL-1β, TNF-α, IL-13 and platelet-derived growth factor (PDGF) [5,6]. Recruitment of macrophages into lung tissue and proliferation of fibroblasts are also induced by TGF-β [7,8]. TGF-β causes an imbalance between production and degradation of extracellular matrix (ECM), resulting in excessive accumulation of ECM in lung interstitium [7,9]. Moreover, TGF-β induces apoptosis of type 2 alveolar epithelial cells, resulting in repeated damage to lung epithelial cells [10]. Myofibroblasts are contractile cells with microfilament bundles and α-SMA, which are the major cells producing ECM in pulmonary fibrosis [7,8,11]. TGF-β induced epithelial–mesenchymal transition (EMT) of lung epithelial cells is recognized to play an important role in myofibroblast formation [12]. Therefore, EMT of alveolar epithelial cells induced by TGF-β may be one of the important mechanisms in the development of pulmonary fibrosis.

Increasing evidence indicates that IL-17 is implicated in pulmonary fibrosis [13]. IL-17 is a cytokine mainly produced by Th17 cells, and TGF-β regulates Th17 differentiation by inducing RORγt. The receptor for IL-17 is present on the surfaces of various cells, including epithelial cells, fibroblasts and granulocytes [13,14,15]. IL-17 has been shown to play an adverse role in pulmonary fibrosis. In IPF patients, IL-17 was localized in the disease sites called fibrotic foci [16]. During the development of bleomycin-induced fibrosis, IL-17 showed a synergistic effect with TGF-β in promoting collagen production [17].

Kurarinone is a prenylated flavonoid component of herbal plants such as *Sophora flavescens*. Kurarinone has the ability to inhibit biological events related to pulmonary fibrosis, such as generation of oxidative stress and production of IL-10, IL-17 and MCP-1 [18,19,20,21,22]. In addition, kurarinone attenuates renal fibrosis via inhibiting the EMT of tubular epithelial cells [23]. Based on these reports, we investigated the role of kurarinone in pulmonary fibrosis using a bleomycin (BLM)-induced pulmonary fibrosis mouse model and explored the associated mechanisms.

## 2. Materials and Methods

### 2.1. Reagents

Flavonoids including kurarinone (Figure S1A) were isolated from *S. flavescens* and purified using the method described by Kwon et al. and Lee et al. [24,25]. Purity was measured at 97.46% by ultra-performance liquid chromatography (UPLC) (Figure S1B). Commercial kurarinone and bleomycin (BLM) used for the in vivo experiment were purchased from Sigma-Aldrich-Merck (St Louis, MO, USA). Pirfenidone (PFD) was obtained from MedChem Express (Monmouth Junction, NJ, USA).

### 2.2. Mice

Male BALB/c mice were purchased from DBL (Eumseing, South Korea) and DO11.10 TCR transgenic mice were obtained from Taconic (Rensselaer, NY, USA). All experimental procedures were approved by the Institutional Animal Care and Use Committee of Korea Research Institute of Bioscience and Biotechnology (approval number: KRIBB-AEC-20008).

### 2.3. Cell Culture

Human BEAS-2B lung epithelial cells were obtained from the American Type Culture Collection (ATCC, Manassas, VA, USA). Cells used in experiments were not used for more than 15 passages. Cells were cultured at 37 °C in a 5% CO_2_-humidified incubator and maintained in high-glucose Dulbecco’s modified Eagle medium (DMEM) containing 10% (*v*/*v*) heat-inactivated fetal bovine serum, 1 mM sodium pyruvate, 2 mM glutamine, 100 U/mL penicillin and 50 μg/mL streptomycin. Cells at a confluence of 60–80% were stimulated with the experimental reagents in serum-free medium as indicated in each experiment. All experiments were replicated three times.

### 2.4. Cell Viability Assay

Cell viability was analyzed using Ez-cytoX (DoGenBio, Seoul, Korea) according to the manufacturer’s protocol and analyses were replicated three times.

### 2.5. Murine Bleomycin-Induced Pulmonary Fibrosis Model

Eight-week old male BALB/c mice (body weights were 22~24 g) were randomly divided into five groups (*n* = 6 in each group): a normal control group (NC), a bleomycin-treated group (BLM), a kurarinone (5 mg/kg) + BLM-treated group (Ku5), a kurarinone (10 mg/kg) + BLM-treated group (Ku10) and a pirfenidone (150 mg/kg) + BLM-treated group (PFD). At day 0, mice were anesthetized using a mixture of ketamine and xylazine by i.p. injection. A total of 50 μL of bleomycin (3 mg/kg body weight, Sigma-Aldrich Inc., St. Louis, MO, USA) was administrated by intratracheal instillation. Sterile saline was administered for the control group instead of bleomycin. Kurarinone solution was prepared by dissolving in the vehicle composed of 20% (*v*/*v*) DMAC (Sigma-Aldrich Inc.), 20% (*v*/*v*) TWEEN80 (Sigma-Aldrich Inc., St. Louis, MO, USA) and 60% (*v*/*v*) HPBCD (Tokyo Chemical Industry, Tokyo, Japan). The indicated dose of kurarinone solution was administered orally five times a week from day 7 to day 27 after bleomycin administration. The NC groups received the vehicle only. On day 28, mice were sacrificed and lung tissues were collected for further analysis after pulmonary function test. Animal experiments were replicated twice.

### 2.6. Pulmonary Mechanical Function Test

Lung mechanics were assessed using a flexiVent system (SCIREQ, Inc., Montreal, QC, Canada) according to the manufacturer’s protocol. Briefly, mice were anesthetized using pentobarbital sodium (Entobar^®^, Hanlim Pharm. Co., Ltd., Seoul, South Korea) to suppress spontaneous breathing. Tracheotomy was performed to insert an 18 G cannula into the trachea to connect the mice with the flexiVent system. Connected mice were ventilated at a respiratory rate of 150 breaths/min and tidal volume of 10 mL/kg against a positive end-expiratory pressure (PEEP) of 3 cmH_2_O with a computer-controlled small-animal ventilator. Measurement of respiratory system mechanics was evaluated assuming four different models. Pressure-derived PV curves were generated to determine the distensibility of the entire respiratory system. Total respiratory system resistance (Rrs), compliance (Crs) and the elastance of the respiratory system (Ers) were measured with the Snapshot-150. G and H, reflecting the damping and elastance of lung tissue, respectively, were obtained from Quick Prime-3. All measurements were conducted in mice with closed chest walls. All data was analyzed using SCIREQ flexiWare (version 7.6, service pack 5, SCIREQ)

### 2.7. Staining for Histopathological Analysis

After sacrificing the mice on day 28, left lung tissues were fixed in 10% formalin, embedded in paraffin and cut into 4 μm thick sections. Sections were stained with hematoxylin and eosin (Sigma-Aldrich Inc., St. Louis, MO, USA) or Sirius Red (Abcam, Cambridge, UK).

### 2.8. Hydroxyproline Assay

The hydroxyproline content of the lung tissues was determined with a Hydroxyproline Colorimetric assay kit (BioVision, Milpitas, CA, USA) according to the manufacturer’s instructions. In brief, lung tissues homogenized in dH_2_O were hydrolyzed with concentrated HCl at 120 °C for 3 h and the supernatants were collected after centrifuging the hydrolyzed homogenates at 10,000× *g* for 3 min. Then, 10 μL of the supernatants was loaded onto a 96-well plate followed by a drying step, after which the samples were assessed for hydroxyproline at 560 nm. The data were expressed as micrograms of hydroxiproline per milligram wet lung weight (μg/mg wet tissue).

### 2.9. Quantitative Real-Time Reverse Transcription-PCR (qPCR)

Total RNA was extracted from cell pellets or homogenized lung tissues. For RNA isolation, the TRIzol reagent (Ambion^®^, Thermo Fisher Scientific, Waltham, MA, USA) was used according to the manufacturer’s protocol. The cDNA was synthesized from 1 μg of total RNA using the ReverTra Ace First Strand cDNA Synthesis Kit (Toyobo, Osaka, Japan). For real-time PCR, the iQ SYBR Green supermix (Bio-Rad, Hercules, CA, USA) and an S1000™ Thermal Cycler (Bio-Rad) were used. Sequences of primers used for qPCR and the accession number of target genes are depicted in Table S1. The relative gene expression levels were evaluated by their ratio to Gapdh mRNA.

### 2.10. Western Blot Analysis

Cell lysates and tissue lysates were harvested with RIPA buffer (Biosesang, Seongnam, South Korea) containing a protease inhibitor cocktail and a phosphatase inhibitor. The proteins were fractionated on 10% SDS-polyacrylamide gels. Gels were transferred to polyvinylidene fluoride membranes, and the membranes were incubated for 1 h in 5% skim milk in TBS-T buffer. Then, membranes were incubated with primary antibodies recognizing pAkt, pSmad2/3, p-ERK, p-p38, p38, ColIα1 (Cell Signaling Technology, Danvers, MA, USA), Akt, Smad2/3 (BD Biosciences, CA, USA), ERK (Santa Cruz Biotechnology, Inc., Dallas, TX, USA), and β-actin (BioLegend, San Diego, CA, USA), followed by incubation with an HRP-conjugated secondary antibody (Jackson ImmunoResearch Laboratories, West Grove, PA, USA). Signals were developed using an enhanced chemiluminescence system (Thermo Fisher Scientific, Waltham, MA, USA). Optical densities of target protein bands were analyzed with ImageJ 1.52a (National Institutes of Health, Bethesda, MD, USA).

### 2.11. Splenocyte Culture and Kurarinone Treatment

Splenocytes were isolated from DO11.10 mice and cultured in RPMI-1640 media with 10% FBS, 50 μM β-mercaptoethanol (Sigma-Aldrich Inc., St. Louis, MO, USA). Then, 500 nM OVA_323~339_ peptide (Peptron, Daejeon, South Korea), 2 ng/mL recombinant murine TGF-β (BioLegend, San Diego, CA, USA) and 10 ng/mL murine IL-6 (BioLegend), which was designated as the Th17 condition, were used to induce differentiation of T cells in splenocytes. The indicated concentration of kurarinone was applied to splenocytes cultured under Th17 conditions for 3 days. Data analysis was based on the results from three replicated experiments.

### 2.12. Measurement of Cytokine Secretion by ELISA

The levels of IL-17 in cell supernatants were analyzed with an IL-17A Mouse Uncoated ELISA Kit (Invitrogen^TM^, Thermo Fisher Scientific) following the manufacturer’s instructions.

### 2.13. Measurement of Total TGF-β1 by ELISA

To prepare lung tissue lysate for ELISA analysis, frozen lung tissues were homogenized with phosphate buffered saline containing 1% Triton X-100 and protease inhibitors. After being centrifuged, the supernatant from the lung homogenate was collected for ELISA analysis. Concentration of TGF-β1 in serum was measured by ELISA kits (R&D System Inc., Minneapolis, MN, USA), according to the manufacturer’s instructions.

### 2.14. Statistical Analysis

Data were analyzed and graphed with GraphPad Prism software (ver. 6.07, GraphPad Software, Inc., San Diego, CA, USA) and are presented as means ± SEMs. Statistical analysis was calculated using analysis of variance (ANOVA) followed by a multiple comparison test with Tukey’s post hoc test. *p* values of less than 0.05 were considered statistically significant.

## 3. Results

### 3.1. Kurarinone Inhibits TGF-β-Induced Epithelial–Mesenchymal Transition in BEAS-2B Cells

**Figure 1:**
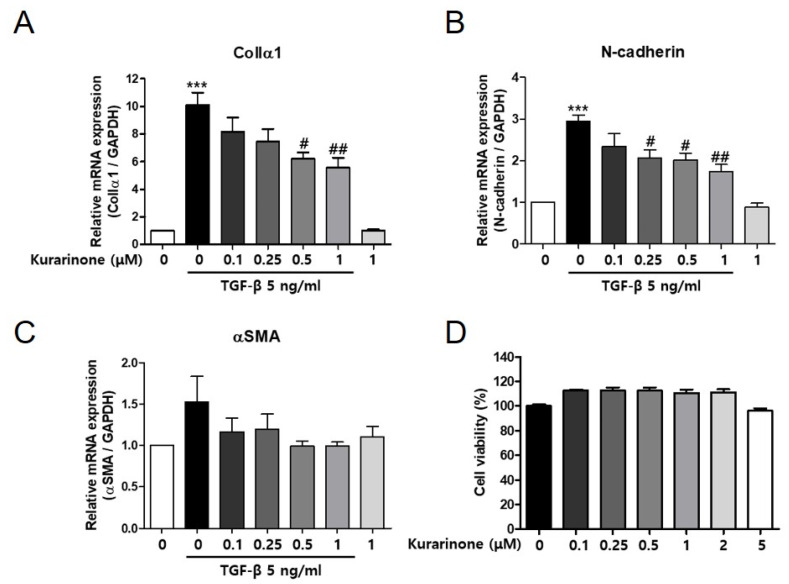
Kurarinone inhibits TGF-β-induced epithelial–mesenchymal transition of alveolar epithelial cell. (**A**) mRNA levels of ColIα1, (**B**) N-cadherin and (**C**) α-SMA in BEAS-2B cells treated with TGF-β (5 ng/mL, 24 h) with or without kurarinone. The mRNA levels were measured by real-time PCR and normalized to *Gapdh* mRNA expression. (**D**) Cell viability of kurarinone-treated BEAS-2B cells was assessed by CCK-8 assay. All experiments were repeated three times and representative results are presented. Statistics are represented as means ± the SEM of each group; *** *p* < 0.001, compared with untreated control, ^#^ *p* < 0.05 and ^##^ *p* < 0.01, compared with TGF-β-treated group.

Epithelial–mesenchymal transition (EMT) of epithelial cells has been shown to be implicated in fibrotic lung diseases. To determine the role of kurarinone in pulmonary fibrosis, we examined the effect of kurarinone on TGF-β-induced EMT in BEAS-2B human lung epithelial cells. TGF-β significantly increased transcript levels of the EMT markers ColIα1 and N-cadherin in BEAS-2B cells (Figure 1A,B). Kurarinone suppressed the TGF-β-induced expression of ColIα1 and N-cadherin in a dose-dependent manner (Figure 1A,B). The expression level of α-SMA in TGF-β-treated BEAS-2B cells was also decreased by kurarinone, albeit below the statistically significant range (Figure 1C). Kurarinone did not affect the viability of the cells in this condition as assessed by CCK-8 (Figure 1D).

### 3.2. Kurarinone Improves the Mechanical Lung Function in Bleomycin-Induced Pulmonary Fibrosis

**Figure 2:**
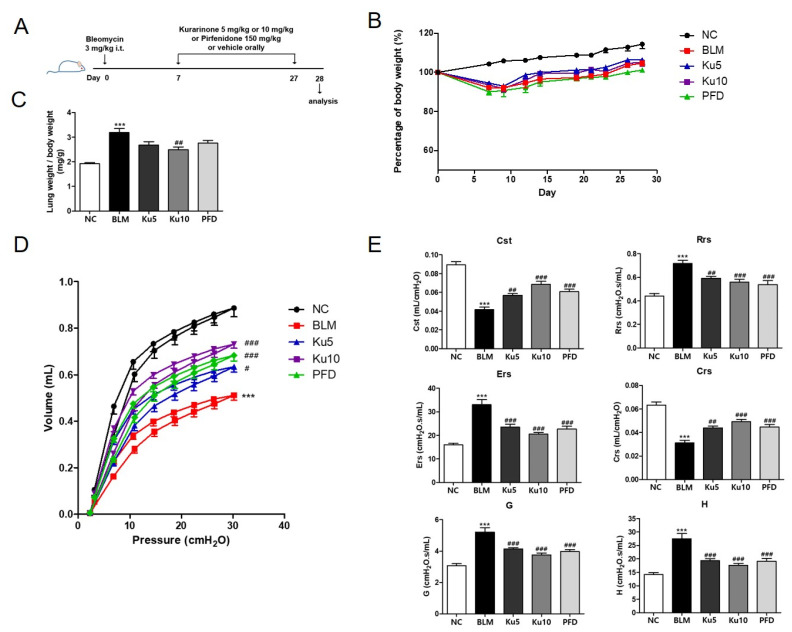
Kurarinone improves mechanical function of lung in bleomycin-induced pulmonary fibrosis model. (**A**) A schematic illustration of BLM administration and drug treatment in this study. (**B**) Relative body weight changes in BLM-challenged mice with or without treatment of kurarinone, as indicated. Mice were randomized into weight-matched groups. The relative body weight was calculated as a percentage of that measured on day 0, which was defined as 100%. Statistics are represented as means ± the SEM of each group per time point (*n* = 6). (**C**) Relative left lung weight in mice challenged with BLM, with or without treatment with kurarinone. Relative left lung weight was calculated as the ratio of left lung weight (mg) to body weight (g) of each mouse. (**D**) Pressure–volume curves show the correlation of the volume of the respiratory system to incrementally increasing pressure. (**E**) The values of Rrs (resistance of the total respiratory system), Ers (elastance of the total respiratory system), Crs (compliance of the total respiratory system), G (damping of tissue), H (elastance of tissue) and Cst (distensibility of the respiratory system) are shown. NC, normal control mice treated with saline only; BLM, BLM-treated mice; Ku5 and Ku10, kurarinone (5 and 10 mg/kg) + BLM-treated mice; PFD, pirfenidone (150 mg/kg) + BLM-treated mice. All data are represented as means ± the SEM (*n* = 6). *** *p* < 0.001, compared with normal control (NC); ^#^ *p* < 0.05, ^##^ *p* < 0.01, ^###^ *p* < 0.001, compared with BLM group.

We next explored the effects of kurarinone on pulmonary fibrosis in vivo using a bleomycin-induced disease mouse model (Figure 2A). The body weight of the mice was reduced by single intratracheal administration of BLM, then recovered gradually after day 7 (Figure 2B). On the other hand, relative lung weight (lung weight/whole body weight) was markedly increased by BLM treatment (Figure 2C). To assess the therapeutic effects of kurarinone, we administered kurarinone from day 7 to day 27 after BLM treatment. Body weight was not significantly changed by kurarinone treatment compared to the BLM-treated group; however, 10 mg/kg of kurarinone alleviated lung weight increase in BLM-treated mice (Figure 2B,C). Oral administration of pirfenidone (PFD), a therapeutic drug for IPF approved by the US FDA, also did not affect the body weight of BLM-treated mice (Figure 2B). PFD also reduced the lung weight increase caused by BLM treatment, albeit with no statistical significance compared to the BLM group (Figure 2C).

The forced oscillation technique (FOT) was used to analyze the effect of kurarinone on the mechanical dynamics of the respiratory system in BLM-treated mice. As shown in the pressure–volume curve, instillation of BLM decreased the volume of the respiratory system significantly (Figure 2D). The distensibility of the respiratory system (Cst) and the compliance of the total respiratory system (Crs) were also reduced by BLM (Figure 2E). The resistance of the total respiratory system (Rrs), the elastance of the total respiratory system (Ers), the damping of tissue (G) and the elastance of tissue were increased in BLM-treated mice (Figure 2E). Oral administration of kurarinone to BLM-treated mice restored the mechanical function of the respiratory system to an extent comparable to PFD (Figure 2D,E). Taken together, these data show that kurarinone restored the impaired mechanical function of the respiratory system through BLM treatment.

### 3.3. Kurarinone Attenuates Fibrotic Changes of Lung Tissues in BLM-Treated Mice

**Figure 3:**
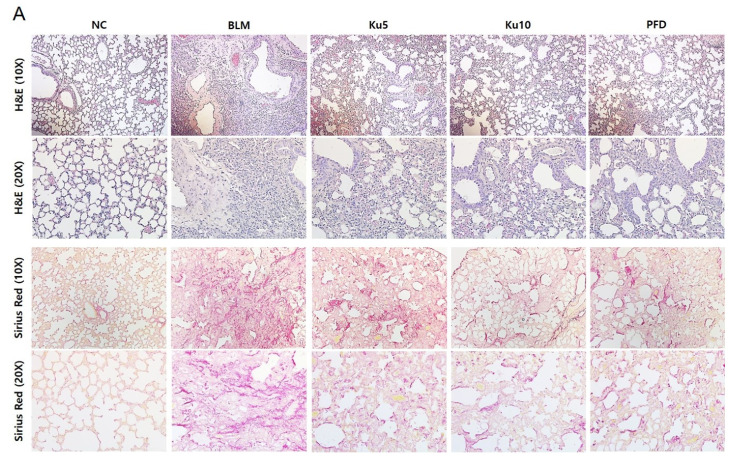

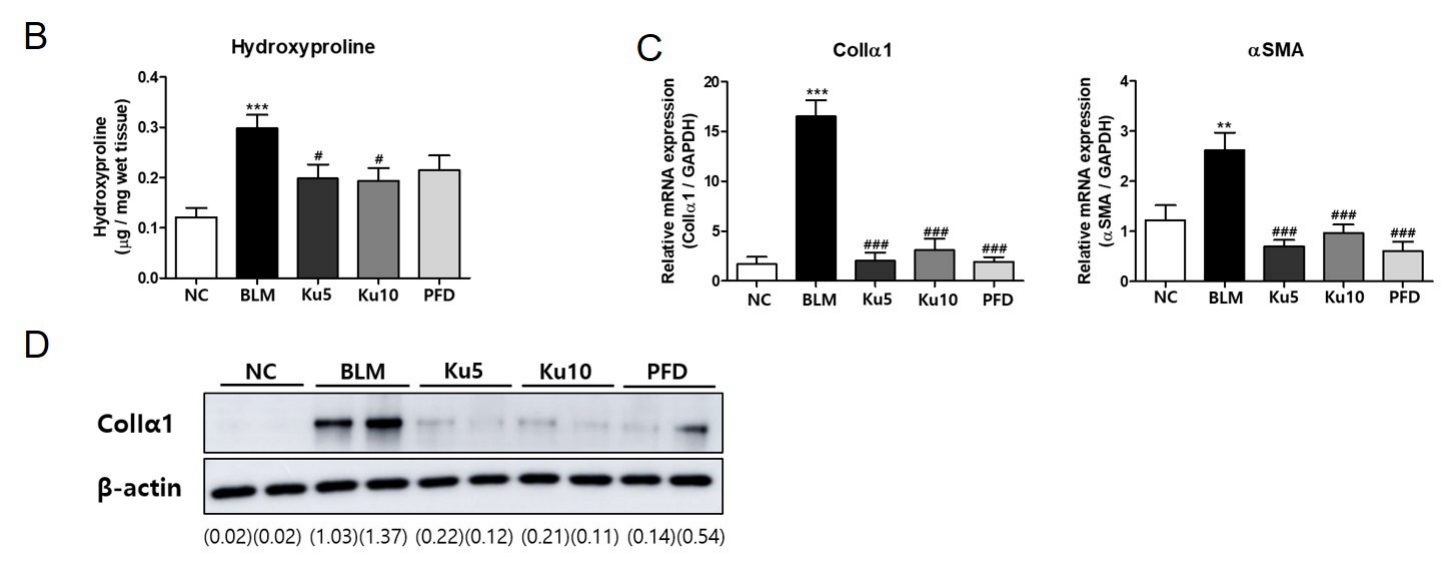
Kurarinone attenuates the fibrotic changes in lung tissue of BLM-induced lung fibrosis mice model. (**A**) Lung tissues were fixed, sectioned at 4 μm and stained with H&E or Sirius Red. (**B**) The amount of hydroxyproline in lung tissues was analyzed as the quantitation of collagen. (**C**) The mRNA levels of ColIα1 and α-SMA in lung tissues were determined by real-time RT-PCR. The mRNA expression data were normalized to *Gapdh* mRNA expression. (**D**) The protein levels of ColIα1 in lung tissues were analyzed by Western blot. The numbers noted below the blotting images indicate the relative band densities of ColIα1, which were calculated and normalized to β-actin. Based on the value of the relative band density, relative protein levels of the target protein were analyzed. Densities of Western blot bands were quantitated using ImageJ. NC, normal control mice treated with saline only; BLM, BLM-treated mice; Ku5 and Ku10, kurarinone (5 and 10 mg/kg) + BLM-treated mice; PFD, pirfenidone (150 mg/kg) + BLM-treated mice. All data are represented as means ± the SEM (*n* = 6). ** *p* < 0.01, *** *p* < 0.001, compared with normal control (NC); ^#^ *p* < 0.05, ^###^ *p* < 0.001, compared with BLM group.

Infiltrated immune cells and fibrotic changes in lung tissues lead to destruction of lung structures and concomitant impairment of mechanical lung functions in pulmonary fibrosis. To evaluate the effect of kurarinone on lung tissues of BLM-treated mice, we performed a histopathological analysis. BLM-treatment increased infiltration of immune cells and destruction of lung structure evidenced by thickening of the alveolar wall and loss of alveolar space (Figure 3A, Upper row). As shown by Sirius Red staining of a histological section of the lung tissue, BLM administration increased the red colored fibers (Figure 3A, Bottom row). Lung collagen contents were increased by BLM treatment (Figure 3B). Kurarinone or PFD treatment attenuated histological changes in lung tissue of BLM-treated mice (Figure 3A). Collagen deposition in lung tissue was also decreased by kurarinone (Figure 3B). To investigate the effects of kurarinone on pro-fibrotic markers, mRNA transcript levels of ColIα1 and α-SMA in the lung were quantified by PCR and the protein level of ColIα1 in lung tissue was analyzed by Western blot. Instillation of BLM significantly increased the mRNA levels of ColIα1 and α-SMA and the amount of ColIα1 protein in lung tissue (Figure 3C). Kurarinone treatment reversed these effects induced by BLM as effectively as PFD (Figure 3C,D and Figure S2). These results indicated that kurarinone has the ability to improve the mechanical function of BLM-treated mice by suppressing fibrotic changes, such as excessive production of extracellular matrix in lung tissues.

### 3.4. Kurarinone Suppressed Phophorylation of Smad2/3 and AKT Mediating the TGF-β Signaling Pathway In Vitro and In Vivo

**Figure 4:**
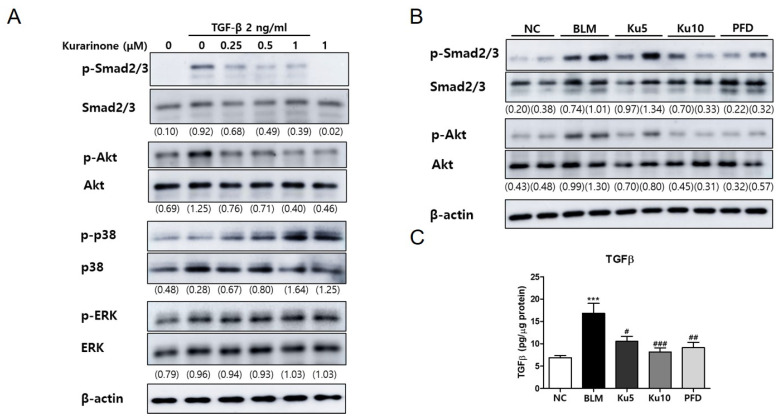
Kurarinone inhibits phosphorylation of Smad2/3 and AKT in TGF-β-treated BEAS-2B cells and lung tissue of a BLM-induced pulmonary fibrosis model. (**A**) Phosphorylation of Smad2/3, AKT, p38 and ERK in BEAS-2B cells treated with TGF-β (2 ng/mL for 30 min) with or without kurarinone was analyzed by Western blot. All experiments were repeated three times and representative results are presented. (**B**) Protein levels of phosphorylated Akt and Smad2/3 in lung tissue were analyzed by Western blot. Optical densities of Western blot bands were quantitated using ImageJ. Numbers noted below the blotting images indicate the relative optical densities of bands of target proteins normalized to the optical density of the total form of each protein. (**C**) Total levels of TGF-β1 in lung tissues sampled from mice of five study groups detected by ELISA. NC, normal control mice treated with saline only; BLM, BLM-treated mice; Ku5 and Ku10, kurarinone (5 and 10 mg/kg, respectively) + BLM-treated mice; PFD, pirfenidone (150 mg/kg) + BLM-treated mice. All data are represented as means ± the SEM (*n* = 6). *** *p* < 0.001, compared with normal control (NC); ^#^ *p* < 0.05, ^##^ *p* < 0.01, ^###^ *p* < 0.001, compared with BLM group.

To investigate the molecular mechanism of the effects of kurarinone on pulmonary fibrosis, phosphorylation of proteins associated with TGF-β signaling were examined. Phosphorylation of Smad2/3 and AKT was significantly increased by TGF-β in BEAS-2B cells, while phosphorylation of ERK and p38 was not significantly increased (Figure 4A and Figure S3). Kurarinone reduced phosphorylation of Smad2/3 and AKT, which was induced by TGF-β (Figure 4A and Figure S3). These results suggested that kurarinone effectively suppressed TGF-β-induced EMT of lung epithelial cells via inhibition of both Smad-dependent and Smad-independent pathways. Importantly, kurarinone also inhibited the phosphorylation of Smad2/3 and AKT in the lung lysates of BLM-treated mice. The inhibitory effects of kurarinone were comparable to those of PFD (Figure 4B and Figure S4). Next, we investigated whether kurarinone inhibits TGF-β production. The level of TGF-β in lung tissues was measured by ELISA. As shown in Figure 3C, kurarinone and pirfenidone efficiently reduced BLM-induced TGF-β1 production. Collectively, these data suggest that the therapeutic effect of kurarinone on BLM-induced pulmonary fibrosis may be achieved by inhibition of TGF-β signaling.

### 3.5. Kurarinone Inhibited Th17 Differentiation in the Lungs of a BLM-Induced Pulmonary Fibrosis Model

**Figure 5:**
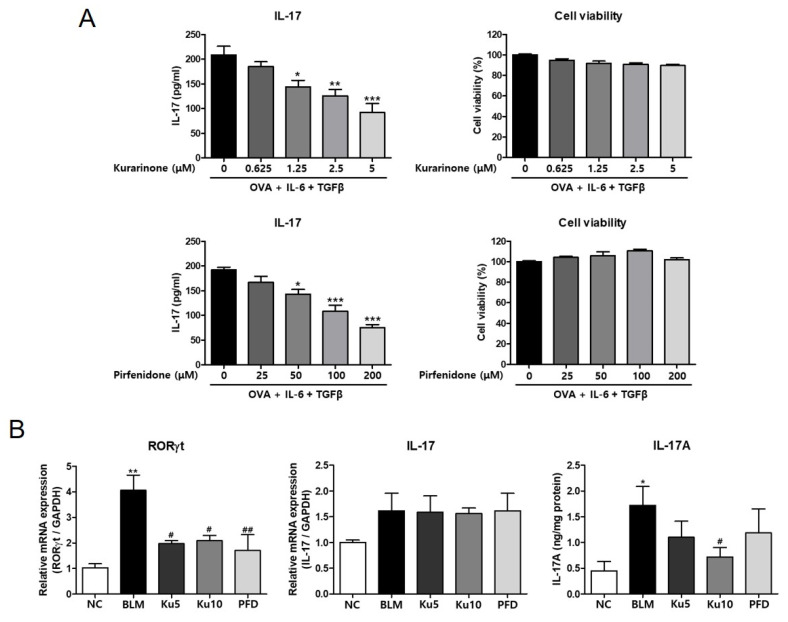
Kurarinone suppresses IL-17 production and the expression of RORγt and IL-17 in the lung tissue of a BLM-induced pulmonary fibrosis model. (**A**) Secreted IL-17 in splenocytes cultured in Th17 conditions with kurarinone or pirfenidone was determined by ELISA. Cytotoxicity of kurarinone or pirfenidone was assessed by CCK-8 assay. All experiments were repeated six times and representative results are presented. Statistics are represented as means ± the SEM of each group; * *p* < 0.05, ** *p* < 0.01, *** *p* < 0.001 compared with the untreated control. (**B**) The transcript levels of RORγt and IL-17 in lung tissues were determined by real-time RT-PCR and ELISA. The mRNA expression data were normalized to *Gapdh* mRNA expression. NC, normal control mice treated with saline only; BLM, BLM-treated mice; Ku5 and Ku10, kurarinone (5 and 10 mg/kg) + BLM-treated mice; PFD, pirfenidone (150 mg/kg) + BLM-treated mice. All data are represented as means ± the SEM (*n* = 6). * *p* < 0.05, ** *p* < 0.01, compared with normal control (NC); ^#^ *p* < 0.05, ^##^ *p* < 0.01, compared with BLM group.

Kurarinone has the ability to inhibit IL-17 production by suppressing Th17 differentiation, which is one of the mechanisms of the pharmaceutical effect of kurarinone on rheumatoid arthritis and experimental autoimmune encephalomyelitis [26]. It has been shown that neutralization of IL-17 attenuated the progress of pulmonary fibrosis [27]. These results suggest that the anti-fibrotic effect of kurarinone might be mediated by suppression of Th17 differentiation or IL-17 production. Thus, we examined the effect of kurarinone on the IL-17 production of differentiating Th17 cells in vitro. Splenocytes from DO 11.10 mice were cultured in a Th17 differentiation condition for 3 days with or without kurarinone. Kurarinone decreased the IL-17 production in the supernatant in a dose-dependent manner within the non-cytotoxic range (Figure 5A, upper panel). The control drug PFD also decreased the IL-17 production (Figure 5A, lower panel). In BLM-induced pulmonary fibrosis, BLM treatment significantly increased the expression of RORγt expression in lung tissue (Figure 5B). Both Kurarinone and PFD suppressed the expression of RORγt mRNA induced by BLM (Figure 5B). Kurarinone and PFD did not significantly alter IL-17 mRNA levels in lung tissue (Figure 5B). However, when we measured the IL-17 protein levels in the lung tissues, we could detect the elevation of IL-17 production by BLM, and 10 mg/kg of kurarinone treatment decreased the BLM-increased IL-17 production.

## 4. Discussion

In this study, we demonstrated that kurarinone suppressed the TGF-β-induced EMT of lung epithelial cells through inhibition of Smad2/3 and AKT signaling. In BLM-induced pulmonary fibrosis, oral administration of kurarinone improved the lung mechanics and respiratory dysfunction. Kurarinone also reduced fibrotic changes, such as collagen accumulation in lung tissue, caused by BLM treatment. The inhibitory effects of kurarinone were achieved by suppressing the activation of Smad2/3 and AKT and down-regulating RORγt in BLM-treated lung tissue.

TGF-β signaling is mediated by two major pathways, Smad-dependent and Smad-independent, with both contributing to TGF-β-induced EMT [7,12,28]. Smad-dependent signaling induces the expression of α-SMA, collagen, PAI-1 and CTGF; Smad-dependent Akt activation causes nuclear translocation of β-catenin, resulting in upregulation of α-SMA [7]. On the other hand, non-Smad signaling pathways activate the PAR6, RhoA and PI3K/Akt pathways, which lead to increases in cell mobility by losing the tight junction, the rearrangement of the cytoskeletal structure and the nuclear translocation of β-catenin [12]. According to our results, it is likely that kurarinone suppresses both Smad-dependent and Smad-independent TGF-β signaling pathways. It is interesting to note that a recent report suggested the direct binding of kurarinone on the Ser 473 site of AKT, which implies that AKT might be the target of kurarinone in suppressing TGF-β signaling [19]. ERK and p38 have been shown to be the targets of the anti-inflammatory effect of kurarinone [19]. However, our results showed that kurarinone did not significantly affect the TGF-β-induced phosphorylation of p38 and ERK in lung epithelial cells. Thus, our data indicated that the inhibitory effect of kurarinone on TGF-β signaling of lung epithelial cells may not mediated by the MAPK signaling pathway.

Currently, the molecular target of kurarinone that accounts for the inhibitory effect of TGF-β signaling has not been identified. Recently, HSP90 has been identified as a pro-fibrotic marker regulating TGF-β signaling. HSP90 was elevated in the lungs of patients with IPF and in a mouse model of pulmonary fibrosis [29,30]. In particular, HSP90 binds with TGF-β receptor 2, inhibits its ubiquitination and degradation by the proteasome and then proceeds to TGF-β downstream signaling, such as Smad2/3, Akt, GSK-3β and ERK phosphorylation [31]. It is important to note a recent report showing that myricetin, a flavonoid commonly found in dietary sources, inhibits Smad2 phosphorylation and MAPK and Akt signaling through interaction with HSP90β and TGF-β receptor 2. The authors of this report also showed that myricetin at a dose of 25–100 mg/kg ameliorated BLM-induced pulmonary fibrosis in mice [32]. Further study will be required to clarify the involvement of HSP90 in the inhibitory effects of kurarinone on TGF-β signaling and pulmonary fibrosis.

In addition to the inhibitory effect on TGF-β-signaling, we found that kurarinone decreased IL-17 production and the expression of RORγt, a key regulator of IL-17 transcription, in vitro and in vivo, respectively. Reduction of IL-17 production in splenocytes cultured in Th17 conditions might be mediated by kurarinone-induced inhibition of AKT activation, which is a regulating mechanism of IL-17 production in differentiating Th17 cells [22,33]. Although mRNA expression of IL-17 in the lung tissue of BLM-induced pulmonary fibrosis was not affected by kurarinone or PFD treatment, kurarinone at a higher dose (10 mg/kg) significantly inhibited IL-17 production at the protein level. At 28 days after BLM treatment, it may have been difficult to detect significant differences in IL-17 expression as the transition from the inflammatory phase to fibrotic changes occurred [34]. On the other hand, we might have been able to detect the differences in IL-17 protein levels because of the longer stability of the protein.

Oxidative stress is an important factor associated with the pathogenesis of IPF [18]. It has been shown that modulators of antioxidant defenses, such as Nrf2, haem oxygenase (HO)-1 and NADPH oxidase (NOX)4, are important for treatment of lung fibrosis. In particular, an important role of Nrf2 in the pathogenesis of IPF has been demonstrated [35,36], and Nrf2 is considered as a promising therapeutic target for IPF [37]. The transcription factor Nrf2 plays a central role in the oxidative stress response pathway. In response to stress, Nrf2 is released from its negative regulator kelch-like ECH-associated protein 1 (KEAP1) and traffics to the nucleus to activate the antioxidant response element (ARE) that controls a series of antioxidant genes. In fact, a number of compounds derived from herbs, such as curcumin, baicalein, triptolide, celastrol and salidroside, have been shown to have inhibitory effects on pulmonary fibrosis through mechanisms involving ROS and Nrf2 [38]. Recently, it was reported that kurarinone activated Nrf2 by downregulation of the expression of KEAP1, leading to the expression of antioxidant enzymes, including HO-1 in human prostate cancer cells [39]. Collectively, these studies suggest that the effect of kurarinone on antioxidant defenses might be an additional molecular mechanism for its inhibitory effect on pulmonary fibrosis.

Through the administration of bleomycin, acute inflammatory responses, such as cytokine production and massive infiltration of immune cells into lung tissue, can be induced, lasting up to 7 days; in turn, fibrogenic changes with the deposition of fibrotic extracellular matrix in the lung structure appear [40]. There have been many reports about the preventive effects of natural compounds that have an ability to protect cells from the cytotoxic effect of fibrosis inducers via anti-inflammatory or anti-oxidative functions [41]. However, the preventive effect of natural compounds does not guarantee therapeutic effects that are beneficial for the treatment pulmonary fibrosis and in prolonging the life span of IPF patients. Therefore, in order to evaluate the therapeutic effect on pulmonary fibrosis, kurarinone was orally injected into the BLM-induced pulmonary fibrosis model from day 7, when the initial inflammatory response was almost completed. As indicated in Figure 2 and Figure 3, kurarinone efficiently restored mechanical lung functions and suppressed fibrotic changes in lung tissue as much as pirfenidone. These results suggest that kurarinone has therapeutic effects on pulmonary fibrosis.

Collectively, we demonstrated that kurarinone has an anti-fibrotic effect that attenuates pulmonary fibrosis via interference with the TGF-β signaling pathway and may be beneficial for IPF patients.

## Data Availability

Not applicable.

## Supplementary Materials

The following are available online at https://www.mdpi.com/article/10.3390/ijms22168388/s1.

## Abbreviations

α-SMA	alpha-smooth muscle actin
BLM	bleomycin
Col1α1	collagen type 1 alpha 1
ECM	extracellular matrix
EMT	epithelial–mesenchymal transition
HPLC	high-performance liquid chromatography
IL-	interleukin-
IPF	idiopathic pulmonary fibrosis
MAPK	mitogen-activated protein kinase
MCP-1	monocyte chemoattractant protein 1
PAI-1	plasminogen activator inhibitor 1
PDGF	platelet-derived growth factor
PFD	pirfenidone
RORγt	PAR-related orphan receptor gamma
TGF-β	transforming growth factor β
TNF-α	tumor necrosis factor α
