# Supplementary material for: Kurarinone Attenuates BLM-Induced Pulmonary Fibrosis via Inhibiting TGF-β Signaling Pathways
Source: Int J Mol Sci. 2021 Aug 4;22(16):8388. doi: 10.3390/ijms22168388 (PMC8395032; doi:10.3390/ijms22168388)
Supplement: Supplementary file 1 [file ijms-22-08388-s001.zip › ijms-1311718-supplementary.pdf]

International Journal of
Molecular Sciences

MDPI

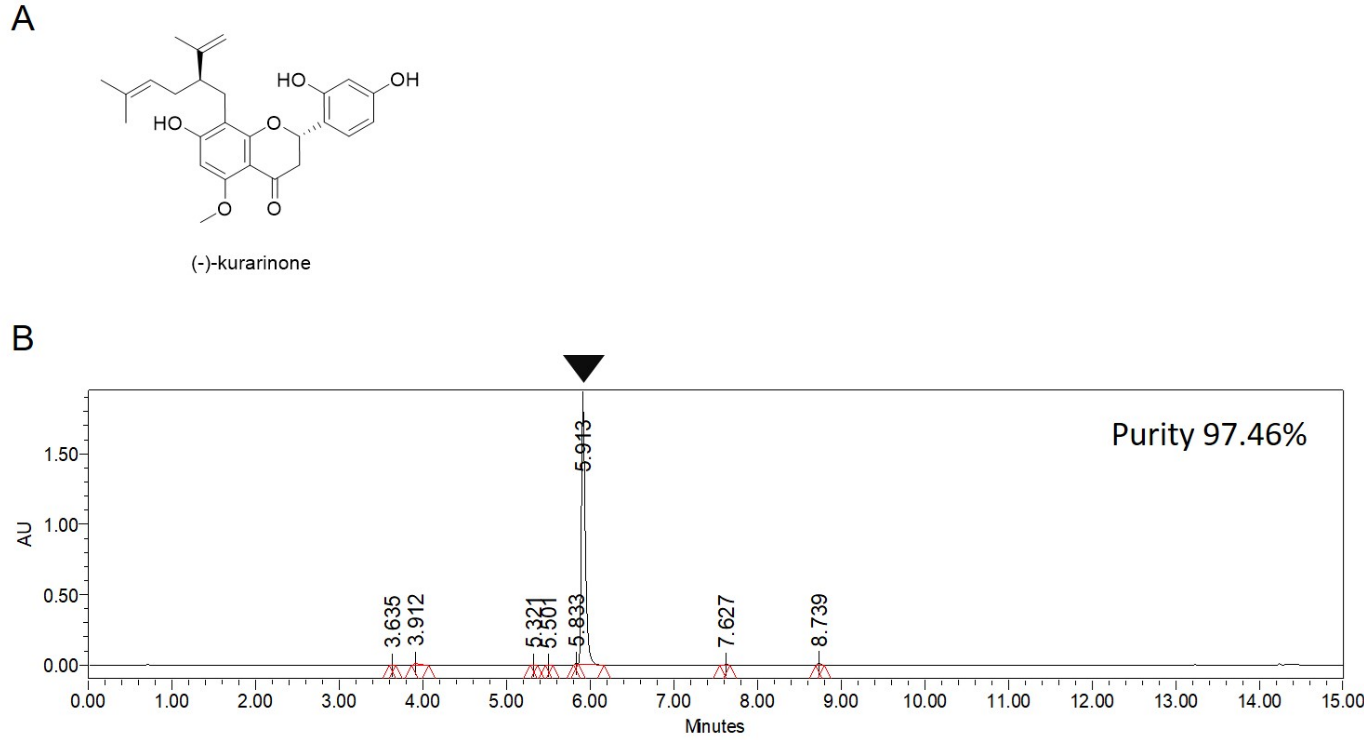

Figure S1. Checking the purity of kurarinone extracted from *Sophora flavescens*. (A) Molecular structure of kurarinone. (B) Purity of extracted kurarinone from *Sophora flavescens* was measured by ultra-performance liquid chromatography (UPLC). Peak indicated with arrowhead is the kurarinone.

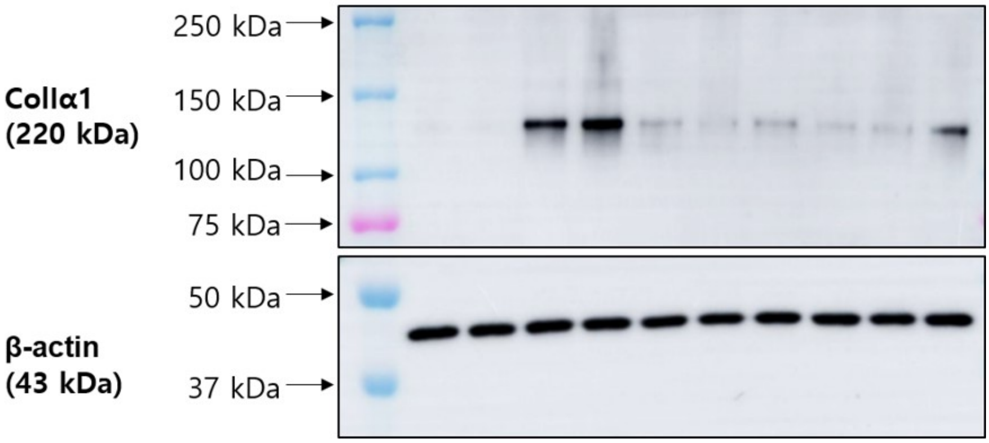

Figure S2. Uncropped western blot images were used for Figure 3. Uncropped western blot images with marker for protein size were used as data for Figure 3. Target proteins were noted on the left side of each membrane images.

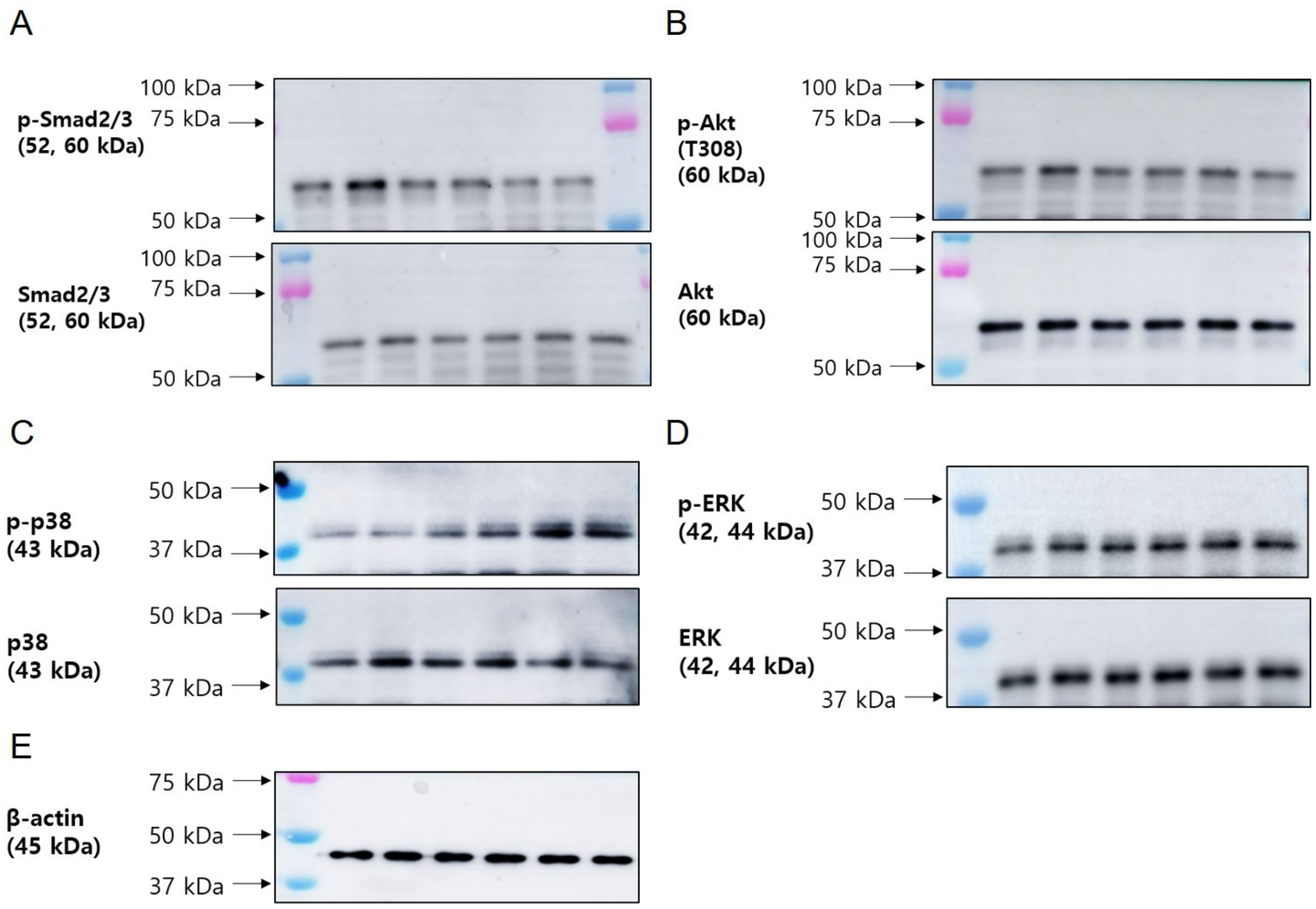

Figure S3. Uncropped western blot images were used for Figure 4A. Uncropped western blot images with marker for protein size were used as data for Figure 4A. Target proteins were noted on the left side of each membrane images.

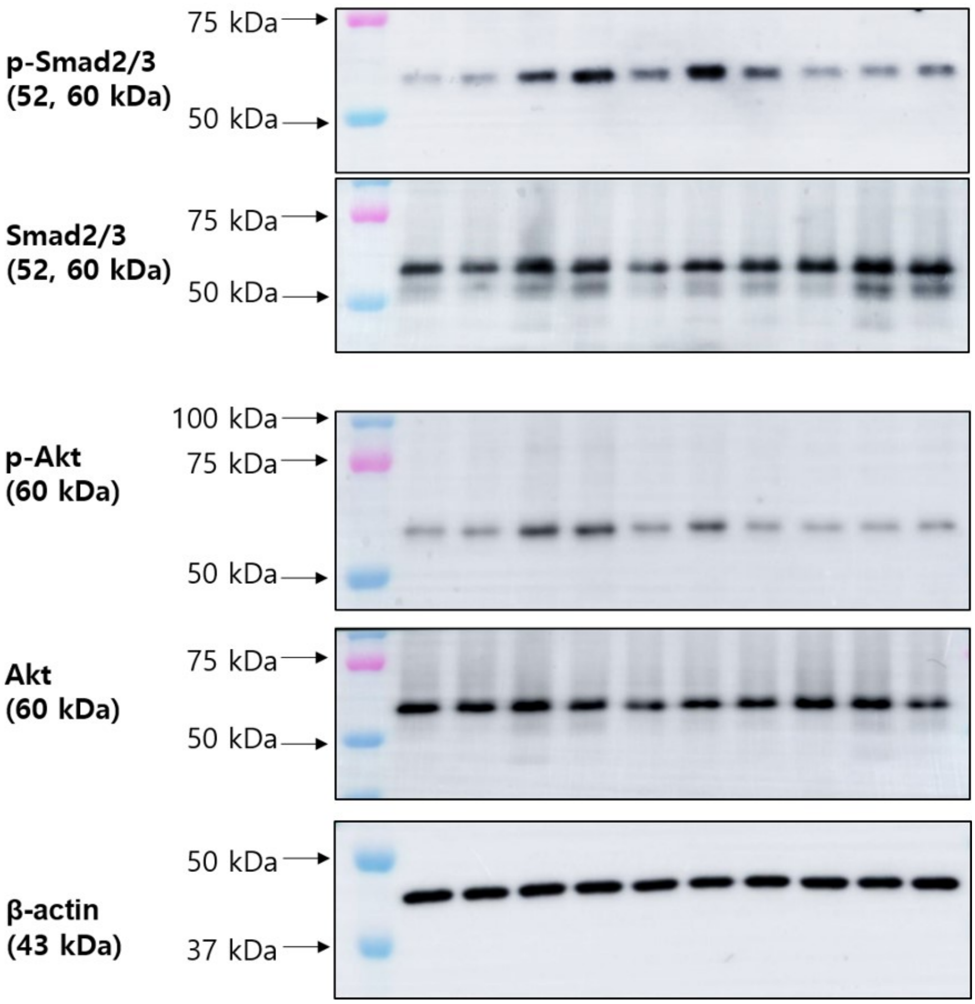

Figure S4. Uncropped western blot images were used for Figure 4B. Uncropped western blot images with marker for protein size were used as data for Figure 4B. Target proteins were noted on the left side of each membrane images.

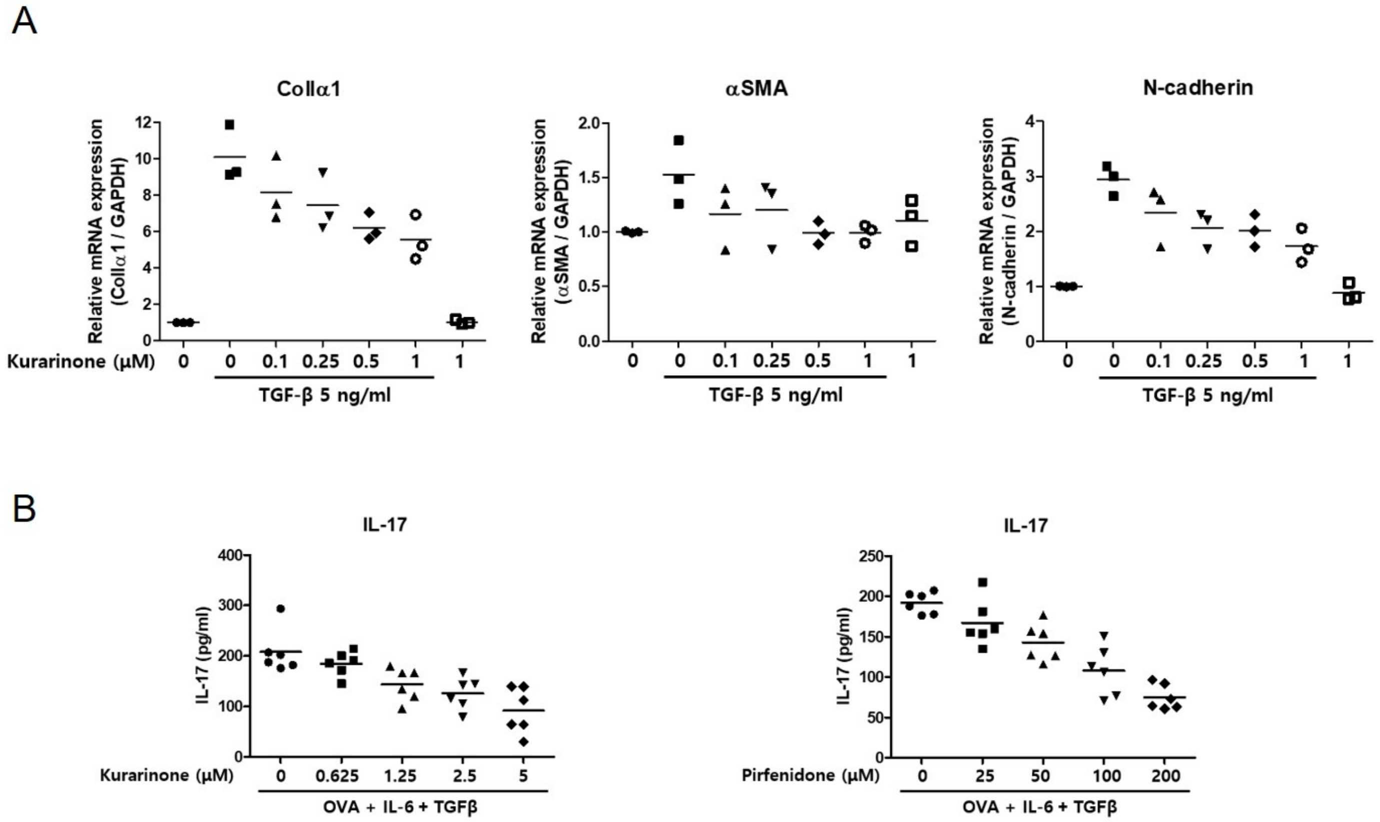

Figure S5. Individual data points to the graphs were used for Figure 1A, 1C, and Figure 5A.

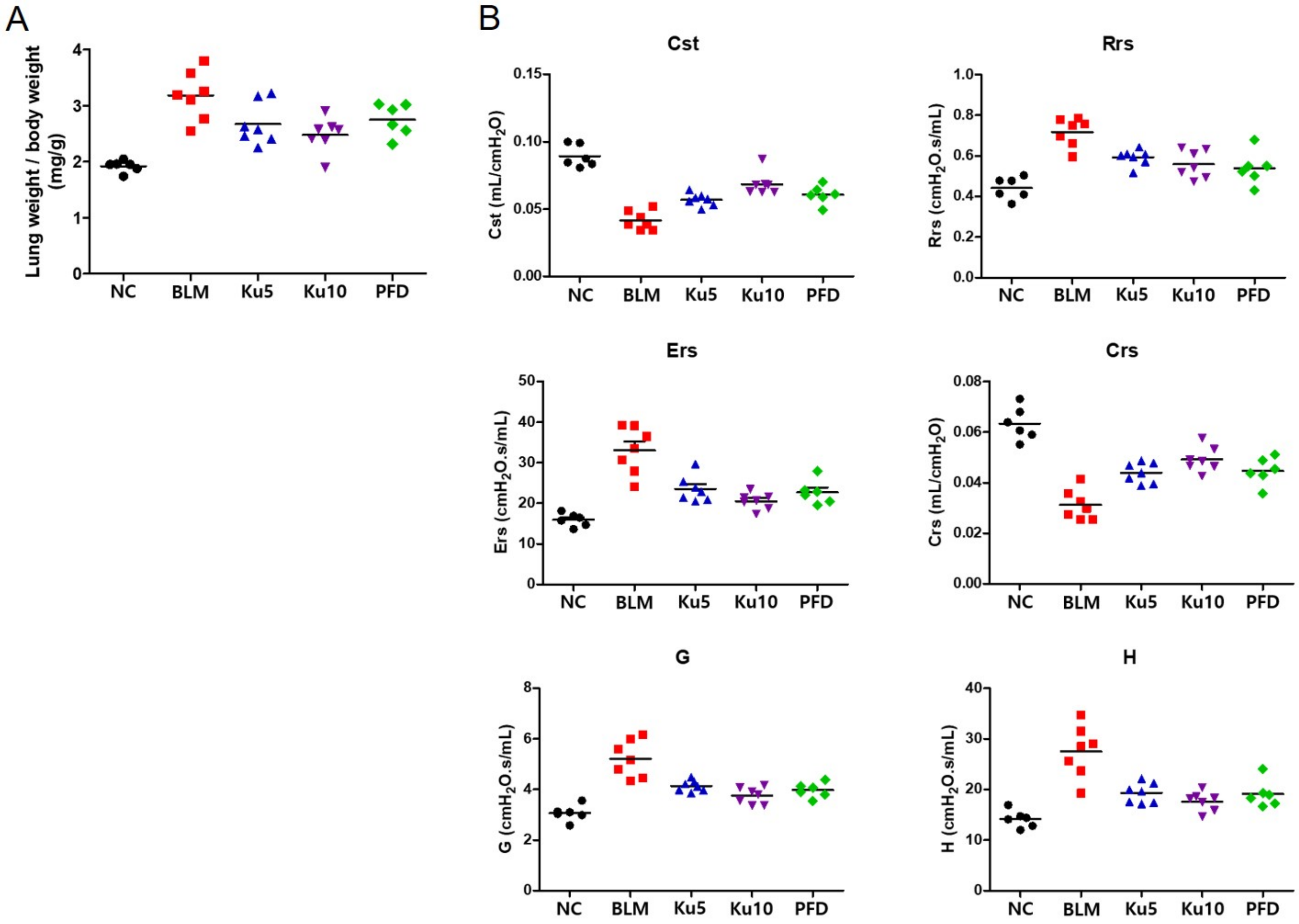

Figure S6. Individual data points to the graphs were used for Figure 2C and 2C.

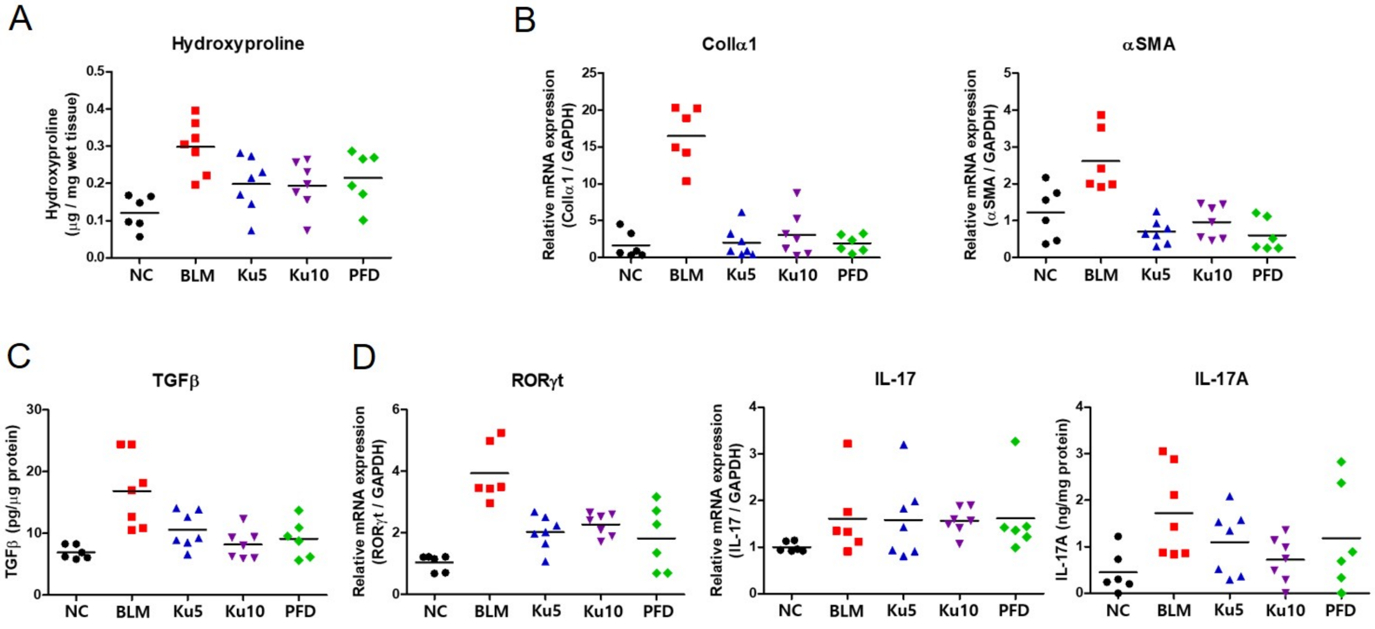

Figure S7. Individual data points to the graphs were used for Figure 3B, 3C, Figure 4C, and Figure 5B.

Table S1. Sequence of primers and accession number of genes.

Genes	Sense (5' to 3')	Antisense (5' to 3')	Acc #
RORγt (mouse)	CGCACCAACCTCTTTTCACG	CAGCTCCACACCACCGTATT	NM_001293734.1
IL-17 (mouse)	CAGCAGCGATCATCCCTCAAAG	CAG GAC CAG GAT CTC TTG CTG	NM_010552
Col1α1 (mouse)	GCAAGAGGCGAGAGAGGTTT	GACCACGGGCACCATCTTTA	NM_007742.4
α-SMA (mouse)	TCCTGACTGAGCGTGGCTA	GTTTCGTGGATGCCCGCTG	NM_007392.3
Col1α1 (human)	AGTGGTTTGGATGGTGCCAA	ACCCTGGGGACCTTCAGAG	NM_000088.3
N-cadherin (human)	GAACTGCAAAGCACCTGTGAG	GTGTAGCTCTCGGCGTCAAA	NM_004360.4
α-SMA (human)	TCCCTGAACACCACCCAGTG	GTGCTTCGTCACCCACGTA	NM_001613.2
